# Clinical and Immunopathologic Profile of Mexican Patients with IgG4 Autoimmune Pancreatitis

**DOI:** 10.5402/2012/164914

**Published:** 2012-05-14

**Authors:** María T. Bourlon, Christianne Bourlon, Yemil Atisha-Fregoso, Fredy Chable-Montero, Marco A. Teliz, Arturo Angeles-Angeles, Eduardo Carrillo-Maravilla, Luis Llorente, Luis F. Uscanga

**Affiliations:** ^1^Department of Internal Medicine, Instituto Nacional de Ciencias Médicas y Nutrición Salvador Zubirán, Vasco de Quiroga 14, 14000 México, DF, Mexico; ^2^Department of Immunology and Rheumatology, Instituto Nacional de Ciencias Médicas y Nutrición Salvador Zubirán, Vasco de Quiroga 14, 14000 México, DF, Mexico; ^3^Department of Radiology, Instituto Nacional de Ciencias Médicas y Nutrición Salvador Zubirán, Vasco de Quiroga 14, 14000 México, DF, Mexico; ^4^Department of Pathology, Instituto Nacional de Ciencias Médicas y Nutrición Salvador Zubirán, Vasco de Quiroga 14, 14000 México, DF, Mexico; ^5^Department of Gastroenterology, Instituto Nacional de Ciencias Médicas y Nutrición Salvador Zubirán, Vasco de Quiroga 14, 14000 México, DF, Mexico

## Abstract

Autoimmune pancreatitis is part of the spectrum of IgG4-associated diseases. Its diagnostic criteria and histological subtypes have been formally proposed recently and although based on current data it has been suggested that there are differences in clinical presentation among populations, more research is needed to properly establish if this heterogeneity exists. In this paper, we describe 15 cases of autoimmune pancreatitis diagnosed at a Mexican centre of reference, all of them associated to the lymphoplasmocytic sclerosing pancreatitis variant. The mean age at the onset of symptoms was 47.5 ± 14.4 years, and 53% of patients were male. The main manifestations were weight loss (87%), obstructive jaundice (53%), and acute (27%) and chronic (27%) pancreatitis. Only 20% of patients had high IgG4 serum levels at the time of diagnosis. All patients receiving prednisone responded favourably, both in their pancreatic and extrapancreatic manifestations. Clinical manifestations of Mexican patients showed certain differences with respect to those usually reported.

## 1. Introduction

Autoimmune pancreatitis has travelled a relatively short way from its first description by Sarles et al. in 1961 [1] to the formal suggestion of the very concept of autoimmune pancreatitis by Yoshida et al. in 1995 [2], and its subsequent inclusion in a new group of diseases with systemic involvement associated with an increase in serum IgG4 levels, after the depiction by Kamisawa et al. [3].

Most descriptions have, up to now, been done on Asian patients, even though there have been descriptions of patients in other geographical areas [4, 5].

In spite of the scant information available, mainly due to the few reported cases of the disease and its aforementioned “short” history, it is possible to assert that there are some differences in the clinical presentation of patients of different ethnicities. In conjunction with the scarcity of available information, there is the added problem that until recently, there is a lack of universally recognised criteria for the diagnosis of the disease. Asian, European, and North American consensus have so far been published [6–8] and most centers use distinct criteria, making difficult to establish comparison among patients in whom the diagnosis was made at different geographical areas. Recently, an international panel of experts met to develop the International Consensus Diagnostic Criteria for Autoimmune Pancreatitis to favor the worldwide recognition of the illness [9].

In this consensus, emphasis is placed on the advantages of classifying autoimmune pancreatitis into either of two types: lymphoplasmocytic sclerosing pancreatitis (LPSP) and idiopathic duct-centric pancreatitis (IDCP), since it has been so far ascertained that there are significant variations in clinical presentation, depending on the histological characteristics of the pancreatitis under study [10].

For this reason, it is important to obtain clinical descriptions of the disease, together with its histopathological characteristics, as this will allow us to lay the foundations for our understanding of the disease behaviour. The aim of this study is to identify and describe the general characteristics of patients diagnosed with autoimmune pancreatitis at a third-level hospital in Mexico.

## 2. Patients and Methods

From our hospital records, we identified 15 patients who met the histological criteria for autoimmune pancreatitis. All patients, but one, were Mexican Mestizos. A Mexican Mestizo is defined as an individual who was born in Mexico and is a descendant from the native inhabitants of such region, and from individuals, mainly Spaniards of white or African origin, or both, who came to America during the 16th century. The other patient was a Colombian.

Laboratory, radiologic, and clinical features were obtained from clinical files.

Total IgG and IgG4 levels were measured by nephelometry. The cut-off was 1868 mg/dL and 135 mg/dL, respectively.

### 2.1. Pancreatic Histology and Immunostaining

Pancreatic and extrapancreatic specimens were assessed by two pathologists. Morphological features on light microscopy were described. Immunohistochemical staining for IgG4 was performed in all cases and was considered positive with >10 IgG4-positive plasma cells per high power field (HPF). Subtype classification as either lymphoplasmocytic sclerosing pancreatitis (LPSP) or idiopathic duct centric pancreatitis (IDCP) was performed according to the 2011 International Consensus Diagnostic Criteria for Autoimmune Pancreatitis [9]. 

### 2.2. Imaging

Available pretreatment computed tomography (CT) scans (*n* = 13) were reviewed by a single radiologist, while in 2 patients only written radiological reports were at hand. Pancreatic enlargement was defined as an increase in size of the gland in the absence of a discrete mass. Mass was defined as a lesion that had different density compared with surrounding pancreatic tissue. Pancreatic duct patency was assessed and extrapancreatic lesions were recorded. 

### 2.3. Response to Steroid Therapy

Biliary and pancreatic duct strictures were considered steroid-responsive if follow-up imaging showed normal biliary tree and pancreatic duct, respectively. Pancreatic mass/enlargement was considered steroid-responsive if abnormality resolved, with posttherapy imaging showing normal-appearing pancreas or pancreatic atrophy. Given the lack of immediate accessibility to serum IgG4 measurements as a marker of disease, we remained without this response criterion.

## 3. Results

Patient's demographic and clinical characteristics at the outbreak of the disease are shown on Table 1. The mean time lapse from the onset of symptoms to diagnosis was 42.5 months.

Patients were followed up at our institute for 34.9 months (range from 2 to 84 months). Throughout this period, 13 patients reported moderate-to-high intensity abdominal pain. Four episodes of acute pancreatitis were reported and four patients were classified as having chronic pancreatitis. Moreover, weight loss was documented in 13 patients, with a mean loss of 13.3 ± 6 kg. None of the patients had inflammatory bowel disease.

The median of total IgG was 2400 mg/dL (range: 1060 mg/dL–4740 mg/dL). The two patients with elevated levels of IgG4 had values of 264 mg/dL and 591 mg/dL, with total IgG of 1060 mg/dL and 1670 mg/dL, respectively, both within normal values of total IgG.

Additional sites of IgG4+ plasma cell infiltration were histopathologically identified in 9 patients, some of them with more than one infiltration site: myofibroblastic tumour (*n* = 3), lymph node infiltration (*n* = 6), palate (*n* = 1), lacrimal gland (*n* = 1), salival gland (*n* = 1), gallbladder (*n* = 2), prostate (*n* = 1), orbit (*n* = 1), liver (*n* = 1), and kidney (*n* = 1).

Nine patients (60%) underwent major abdominal surgery (biliodigestive derivation or Whipple procedure).

### 3.1. Imaging

Radiological data before treatment were available for review in 13 patients, for the other 2 cases only written radiological reports were analyzed. Twelve patients had evidence of pancreatic enlargement as previously defined, 3 of this cases were interpreted as having a mass. Pancreatic duct dilatation was evidenced in 5 cases. In 4 cases, biliary tract dilatation was observed. The most common extrapancreatic involvement identified was abdominal lymphadenopathy in 5/15 cases, followed by interstitial lung involvement in 4/15 cases, and finally 3/15 patients had evidence of glandular involvement on head CT.

### 3.2. Pathology

Glandular organs, including pancreas, lachrymal glands, and minor and major salivary glands, showed predominantly acinar atrophy and a variable degree of interstitial and periductal inflammatory infiltrate with abundant plasma cells and fibrosis. Lymph nodes, including peripancreatic ones, had follicular hyperplasia and dilated sinuses with abundant plasma cells. All cases met criteria for LPSP (type 1).

### 3.3. Response to Treatment

A total of 8 patients were treated with steroids. All of them received oral prednisone at doses of at least 20 mg/qd. All patients responded to treatment and, to this day, none has relapsed although this is not yet significant as the follow-up time from the beginning of treatment has been brief due to delay in diagnosis establishment.

## 4. Discussion

As was previously mentioned, two main histopathological variants of autoimmune pancreatitis are currently recognised, and they behave clinically in quite different manners. The recently established International Consensus has suggested the use of this classification, but this is not, however, always feasible due to the need of a sufficiently large sample of tissue for a pathologist to study.

In this work, we introduce the first series of cases of autoimmune pancreatitis in the Mexican population, all of them confirmed by histopathological analysis.

The review of these cases showed that all patients corresponded to the LPSP variant, which is the most frequently reported, even though our patients' mean age was much lower than that expected for this histological type (mean age of 61.6 versus 47.5 for out population). Nowadays, it is recognised that one of the most common symptoms is obstructive jaundice [11]; however, we found it with a lower frequency than that previously reported, since in Asian patients the frequency goes from 74 to 88% [12, 13], whereas only 53% of our patients had this symptom at the onset of the disease. It is worth remarking that we found a relatively high frequency of acute pancreatitis, which in a recent multicentre study was mainly associated to IDCP [6]. Abdominal pain was also reported with a higher frequency than expected according to previous reports [14].

Extrapancreatic involvement has been mainly associated to LPSP with, in some cases, 2 to 4 organs affected [15]. This is congruent with our findings, as we found 9/15 patients with multiorgan involvement, although organ involvement could probably be more common but pass unnoticed for not making a direct search of it.

It is important to point out that, due to the clinical presentation of this disease, the differential diagnosis is established mainly with pancreas cancer. In this regard, even though pancreatic mass was identified in only 3 patients, pancreatic growth in the imagining study was found in 12 patients which, in conjunction with the weight loss in the majority, gave rise to a high rate of surgeries (biliodigestive derivation or Whipple procedure).

In order to avoid this, one of the main elements that hint at a diagnosis of autoimmune pancreatitis before the surgery alternative is the increase in serum IgG4, which has been found to be high in 75% of patients in some series [16, 17]. In this concern, there are two important points in our series: firstly, IgG4 measurement was requested in only 10/15 patients, which indicates the low suspicion levels prevalent with regard to the disease, something that we hope will change soon; secondly, that only 20% of our patients showed an increase in serum IgG4 levels, which goes against what has been reported up to now. There is no clear explanation for this. The search for cases was based on histopathological results, and perhaps if a systematic measurement of IgG4 serum levels in patients suspected of harboring the disease had taken place, a greater prevalence of high IgG4 would have been found. Be this as it may, only a systematic study of this pathology within our ethnic group can really answer whether these observations are truly an atypical manifestation. It is also worth mentioning that Kamisawa et al. [18] recently published the clinical characteristics of patients with IgG4-negative autoimmune pancreatitis, in which there were differences with respect to those patients that showed an increase, and these patients seem closer to the presentation of our cases.

In conclusion, autoimmune pancreatitis is a disease that is currently undergoing a process of definition of its clinical and diagnostic characteristics. The first cases are being found within our population, and a greater body of evidence is still being needed in order to establish clearly its presentation mode. Notwithstanding this, this series results seem to indicate that, within the Mexican population, patients with autoimmune pancreatitis with LPSP are affected from the disease at an earlier age, with a low percentage rate for high IgG4 serum levels, and with the main symptoms being abdominal pain, pancreatitis, weight loss, and obstructive jaundice.

## Figures and Tables

**Table 1:** Patient's demographic and clinical characteristics at the outbreak of the disease.

Age (mean ± SD) years	47.5 ± 14.4 (16–69) years.
Gender (male)	8 (53%)
Family history of autoimmune diseases	3 (20%)
Initial manifestations	
Weight loss	13 (87%)
Abdominal pain	9 (60%)
Bile duct stenosis/obstructive jaundice	8 (53%)
Palpebral or periorbital swelling	2 (13%)
Recurrent acute pancreatitis	2 (13%)
Salivary gland swelling/Xerostomy	1 (7%)
Pancreatic focal swelling	1 (7%)
Other autoimmune diseases	2 (13%)*
Increased serum IgG4 (>135 mg/dL)	2/10
Rheumatoid factor	4/8
Positive antinuclear antibodies	6/9
Diabetes mellitus	3/15
Increased IgG	5/10

*Sjögren's syndrome and Graves' disease.

## References

[1] Sarles H, Sarles JC, Muratore R, Guien C (1961). Chronic inflammatory sclerosis of the pancreas: an autonomous pancreatic disease?. *The American Journal of Digestive Diseases*.

[2] Yoshida K, Toki F, Takeuchi T, Watanabe SI, Shiratori K, Hayashi N (1995). Chronic pancreatitis caused by an autoimmune abnormality. Proposal of the concept of autoimmune pancreatitis. *Digestive Diseases and Sciences*.

[3] Kamisawa T, Funata N, Hayashi Y (2003). A new clinicopathological entity of IgG4-related autoimmune disease. *Journal of Gastroenterology*.

[4] Takuma K, Kamisawa T, Igarashi Y (2011). Autoimmune pancreatitis and IgG4-related sclerosing cholangitis. *Current Opinion in Rheumatology*.

[5] Kamisawa T, Chari ST, Giday SA (2011). Clinical profile of autoimmune pancreatitis and its histological subtypes: an international multicenter survey. *Pancreas*.

[6] Okazaki K, Kawa S, Kamisawa T (2006). Clinical diagnostic criteria of autoimmune pancreatitis: revised proposal. *Journal of Gastroenterology*.

[7] Kim KP, Kim MH, Kim JC, Lee SS, Seo DW, Lee SK (2006). Diagnostic criteria for autoimmune chronic pancreatitis revisited. *World Journal of Gastroenterology*.

[8] Otsuki M, Chung JB, Okazaki K (2008). Asian diagnostic criteria for autoimmune pancreatitis: consensus of the Japan-Korea Symposium on Autoimmune Pancreatitis. *Journal of Gastroenterology*.

[9] Shimosegawa T, Chari ST, Frulloni L (2011). International consensus diagnostic criteria for autoimmune pancreatitis: guidelines of the international association of pancreatology. *Pancreas*.

[10] Chari ST, Klöeppel G, Zhang L, Notohara K, Lerch MM, Shimosegawa T (2010). Histopathologic and clinical subtypes of autoimmune pancreatitis: the Honolulu consensus document. *Pancreas*.

[11] Sugumar A, Chari ST (2011). Autoimmune pancreatitis. *Journal of Gastroenterology and Hepatology*.

[12] Kamisawa T, Okamoto A (2008). IgG4-related sclerosing disease. *World Journal of Gastroenterology*.

[13] Chari ST, Takahashi N, Levy MJ (2009). A diagnostic strategy to distinguish autoimmune pancreatitis from pancreatic cancer. *Clinical Gastroenterology and Hepatology*.

[14] Deshpande V, Gupta R, Sainani N (2011). Subclassification of autoimmune pancreatitis: a histologic classification with clinical significance. *American Journal of Surgical Pathology*.

[15] Kamisawa T, Takuma K, Egawa N, Tsuruta K, Sasaki T (2010). Autoimmune pancreatitis and IgG4-related sclerosing disease. *Nature Reviews Gastroenterology and Hepatology*.

[16] Ghazale A, Chari ST, Smyrk TC (2007). Value of serum IgG4 in the diagnosis of autoimmune pancreatitis and in distinguishing it from pancreatic cancer. *American Journal of Gastroenterology*.

[17] Hamano H, Kawa S, Horiuchi A (2001). High serum IgG4 concentrations in patients with sclerosing pancreatitis. *New England Journal of Medicine*.

[18] Kamisawa T, Takuma K, Tabata T (2011). Serum IgG4-negative autoimmune pancreatitis. *Journal of Gastroenterology*.

